# Platelets in Dry Eye Disease: A Narrative Review of Biological Mechanisms and Therapeutic Applications Derived from Platelets

**DOI:** 10.3390/life15111785

**Published:** 2025-11-20

**Authors:** Marco Zeppieri, Caterina Gagliano, Alessandro Avitabile, Antonino Maniaci, Francesco Cappellani, Riccardo Foti, Giosuè Giordano Incognito, Dalila Incognito, Roberta Foti

**Affiliations:** 1 Department of Ophthalmology, University Hospital of Udine, 33100 Udine, Italy; 2 Department of Medicine, Surgery and Health Sciences, University of Trieste, 34100 Trieste, Italy; 3 Department of Medicine and Surgery, University of Enna “Kore”, 94100 Enna, Italyrobertafoti@hotmail.com (R.F.); 4 Eye Center “G.B. Morgagni-DSV”, 95125 Catania, Italy; 5 Faculty of Medicine, University of Catania, 95123 Catania, Italy; 6 Plastic Surgery, Department of Surgical Sciences, University of Rome “Tor Vergata”, 00133 Rome, Italy; 7 Obstetrics and Gynecology Unit, Maternal Child Department, Garibaldi Nesima Hospital, 95100 Catania, Italy; 8 Medical Oncology Unit, Department of Human Pathology “G. Barresi”, University of Messina, 98100 Messina, Italy; 9 Division of Rheumatology, A.O.U. “Policlinico-San Marco”, 95123 Catania, Italy

**Keywords:** platelets, dry eye disease, ocular surface, regeneration, inflammation

## Abstract

Background: Platelets have conventionally been viewed as cellular fragments crucial for hemostasis; nonetheless, their extensive secretome of cytokines and growth factors has been increasingly acknowledged as a significant regulator of inflammation and tissue healing at the ocular surface. Aims: The objective of this narrative review is to synthesize existing knowledge of platelet biology with new findings about the therapeutic use of platelet-derived products in dry eye disease (DED). Methods: A qualitative review of the PubMed, Scopus, and Web of Science databases up to June 2025 identified preclinical, translational, and clinical studies assessing platelet-rich plasma (PRP), plasma rich in growth factors (PRGF), platelet lysate, and autologous serum tears for dry eye disease (DED) and associated ocular surface disorders. Results: Platelet-derived formulations have exhibited reliable immunomodulatory and regenerative effects by diminishing inflammatory signaling, lowering cytokine expression, and facilitating epithelial and neurotrophic restoration. Clinical investigations have indicated enhancements in tear film stability, corneal staining, and patient-reported symptoms, especially in cases of moderate-to-severe or refractory illness. Nonetheless, methodological diversity, inconsistent preparation techniques, and restricted sample sizes have impeded comparability among experiments. Conclusions: Platelet-derived treatments constitute a biologically viable and clinically promising strategy for the management of dry eye disease (DED). Future research must emphasize the standardization of preparation protocols, the identification of predictive biomarkers such as transforming growth factor-β1 (TGF-β1), nerve growth factor (NGF), and matrix metalloproteinase-9 (MMP-9), as well as the design of multicenter randomized controlled trials to guarantee reproducible, GMP-compliant clinical applications.

## 1. Introduction

Dry eye disease (DED) is acknowledged as a multifactorial condition affecting the tear film and ocular surface, resulting in pain, visual impairment, and possible damage to the ocular surface [1,2,3]. The TFOS DEWS II study states that dry eye disease (DED) arises from the disruption of tear film homeostasis, characterized by hyperosmolarity, inflammation, and neurosensory dysfunctions [4]. The estimated prevalence ranges from 5% to 50%, influenced by diagnostic criteria, geography, and environmental factors, with increased frequency observed in women and older persons [5,6]. The disease presents a significant economical and quality-of-life burden, akin to other chronic ocular diseases such as glaucoma or macular degeneration [7,8].

The pathogenesis of dry eye disease (DED) is marked by tear film breakdown, desiccating stress, and the activation of inflammatory pathways involving interleukins, tumor necrosis factor-α, and matrix metalloproteinases, which compromise epithelial barrier integrity and lead to goblet cell depletion [9,10,11]. Chronic inflammation and hyperosmolarity create a self-sustaining “vicious cycle” that perpetuates epithelium apoptosis, diminishes corneal sensitivity, and modifies the neural control of lacrimal gland secretion [12,13,14]. These pathways lead to persistent eye pain, variable vision, and, in severe instances, irreversible epithelium damage and nerve degeneration.

Traditional therapy approaches—such as artificial tears, anti-inflammatory eye drops, punctal occlusion, and immunomodulators like cyclosporine A and lifitegrast—have mitigated symptoms but seldom restore normal epithelium structure or neurotrophic equilibrium [15,16,17]. Prolonged corticosteroid use also poses hazards of increased intraocular pressure and cataract development. As a result, there is increasing interest in physiologically based therapies that can facilitate authentic tissue regeneration and immunological homeostasis, rather than only providing symptomatic alleviation [18,19].

Platelets have become essential mediators of wound healing and immune modulation by secreting cytokines, chemokines, and growth factors such as platelet-derived growth factor (PDGF), transforming growth factor-β (TGF-β), epidermal growth factor (EGF), nerve growth factor (NGF), and vascular endothelial growth factor (VEGF) [20,21,22]. This biological repertory justifies the utilization of platelet-derived preparations—such as platelet-rich plasma (PRP), plasma rich in growth factors (PRGF), platelet lysate, and autologous serum tears—as regenerative and anti-inflammatory treatments for ocular surface disorders [23,24,25,26,27,28]. Growing preclinical and clinical evidence indicates that these autologous biologics can expedite epithelial closure, regulate immunological activation, and promote corneal nerve regeneration in moderate-to-severe dry eye disease (DED) [29,30,31,32]. This narrative review aims to synthesize and critically evaluate current data on platelet-mediated mechanisms and therapeutic applications in dry eye disease (DED), emphasizing translational challenges, methodological variability, and future research priorities for standardized and evidence-based implementation.

## 2. Materials and Methods

This article serves as a narrative review that synthesizes molecular, translational, and clinical findings about platelet-derived therapeutics in dry eye disease (DED). A structured yet non-systematic search method was employed to achieve extensive research coverage without formal quantitative aggregation, and the review did not comply with PRISMA principles. Electronic searches were performed on PubMed, Scopus, and Web of Science utilizing a combination of Medical Subject Headings (MeSH) and free-text terms as follows: (“platelet-rich plasma” OR “PRP” OR “platelet lysate” OR “plasma rich in growth factors” OR “PRGF” OR “autologous serum tears”) AND (“dry eye disease” OR “DED” OR “ocular surface” OR “ocular surface disorder”). Only peer-reviewed articles in English presenting original preclinical, translational, or clinical findings on platelet-derived products in ocular surface disease were included. Conference abstracts, case reports lacking quantitative endpoints, and narrative or systematic reviews were removed. Key articles’ reference lists were examined to locate further pertinent studies. The variability in study designs and platelet production techniques necessitated a qualitative synthesis of the evidence instead of a meta-analysis.

## 3. Integration of Evidence

The following section integrates findings from preclinical, translational, and clinical investigations to elucidate the immunomodulatory and reparative functions of platelets in DED. By combining mechanistic insights with therapeutic outcomes, this synthesis aims to provide a cohesive understanding of how platelet-derived factors influence ocular surface immunity, epithelial repair, and tear film homeostasis. The discussion is organized to maintain conceptual continuity—beginning with the biological foundations of platelet activity, progressing through experimental and regenerative mechanisms, and culminating in clinical evidence related to platelet-derived preparations.

### 3.1. Platelet Biology and Ocular Surface Immunity

The role of platelets in the immune regulation of the ocular surface has gained considerable attention in the past decade. While their classical function in coagulation and tissue repair is well established, emerging evidence reveals their active contribution to immune homeostasis and modulation of chronic inflammation—key mechanisms in DED pathophysiology [19]. Upon activation, platelets release a complex secretome from their α-granules and dense granules, comprising more than 300 bioactive molecules. These include pro- and anti-inflammatory cytokines (IL-1β, IL-6, IL-10, TGF-β), chemokines (CXCL4, CCL5), and lipid mediators such as platelet-activating factor (PAF), all of which play important roles in orchestrating immune responses [20].

In DED, chronic activation of the innate immune system results in the release of pro-inflammatory cytokines and proteases that damage the epithelial barrier and reduce goblet cell density. Platelet-derived cytokines, including IL-10 and TGF-β, inhibit pro-inflammatory pathways such as NF-κB activation, while concurrently facilitating epithelial regeneration. This coordinated immune-regenerative mechanism underpins the therapeutic advantages noted with PRP formulations in chronic ocular surface inflammation [21,22,23].

Preclinical data derived from various animal models of corneal inflammation and epithelial damage have provided valuable insight into the immunoregulatory effects of platelet-derived formulations. Topical application of PRP or platelet lysate has been associated with a marked reduction in the infiltration of inflammatory cells into corneal and conjunctival tissues, including neutrophils and monocyte-derived macro-phages, typically identified by markers such as CD45 and CD68. In parallel, a downregulation of key pro-inflammatory cytokines—such as interleu-kin-6 (IL-6), tumor necrosis factor-alpha (TNF-α), and monocyte chemoattractant protein-1 (MCP-1)—has been observed following treatment, suggesting attenuation of both local innate immune activation and downstream inflammatory signaling cascades. These effects are thought to be mediated by bioactive components within the platelet secretome, including transforming growth factor-beta (TGF-β), interleukin-10 (IL-10), and a variety of chemokine antagonists.

Moreover, histopathological analyses from experimental studies have demonstrated improved epithelial organization, reduced stromal edema, and decreased matrix metalloproteinase (MMP) activity after platelet-based treatment. Together, these findings support the role of platelet-derived therapies in modulating immune responses on the ocular surface and promoting a microenvironment conducive to epithelial regeneration and homeostasis [24,25,26]. Interestingly, PRP also has a dual effect. While suppressing damaging inflammation, it simultaneously promotes wound healing and epithelial cell proliferation, likely through its growth factor content (EGF, PDGF, VEGF), indicating a coordinated immune-regenerative mechanism [27].

This duality becomes particularly significant in immune-mediated dry eye diseases, such as those associated with Sjögren’s syndrome or graft-versus-host disease (GvHD), where chronic inflammation and delayed tissue repair coexist. Recent evidence from a pilot study supports the use of umbilical-cord-blood-derived products in patients with autoimmune systemic syndromes and severe ocular surface inflammation. Treatment with umbilical cord blood serum led to marked reductions in pain and inflammation and promoted complete corneal healing in a substantial proportion of patients with corneal ulcers unresponsive to standard therapies [28]. Beyond cytokines, platelets also contribute to tissue-level immune surveillance. They interact directly with monocytes, neutrophils, and dendritic cells, modulating their trafficking and antigen-presenting capacity. This modulation occurs not only via secreted factors but also through direct cell-to-cell contact, mediated by P-selectin and CD40L expression [29,30].

Moreover, platelets may affect ocular surface-resident lymphoid tissue, although this mechanism is still under investigation. The possibility that platelet-derived microvesicles carry functional mRNA and miRNA into epithelial and immune cells adds a new layer of post-transcriptional immune regulation, which could be critical in long-term modulation of inflammation in chronic DED [31,32]. Clinically, the anti-inflammatory effects of PRP have been confirmed in multiple studies. Patients with moderate-to-severe DED treated with PRP eye drops reported reduced symptoms and showed decreased conjunctival hyperemia, improved TBUT, and reduced ocular surface staining, all markers of immunological improvement [33,34].

The evidence supports the concept that platelets are active immune mediators in the ocular surface microenvironment. By attenuating innate immune activation, promoting anti-inflammatory cytokine release, and reestablishing epithelial-immune cross-talk, platelet-derived treatments represent a novel class of immune-regenerative therapies in DED (Table 1).

### 3.2. Mechanisms of Epithelial Repair and Tear Film Stabilization

The ocular surface epithelium is a dynamic structure that maintains the interface between the eye and the external environment [35]. In DED, this barrier is often compromised due to a combination of tear film instability, inflammation, mechanical shear stress, and epithelial cell dysfunction. Over time, the damage can become self-perpetuating, leading to chronic symptoms, epithelial breakdown, and delayed wound healing [36]. PRP is used as regenerative option aimed at restoring epithelial structure and function. These preparations are enriched with a broad spectrum of growth factors and signaling molecules released from platelet alpha granules upon activation. Among them, epidermal growth factor (EGF), platelet-derived growth factor (PDGF), transforming growth factor-beta (TGF-β), fibroblast growth factor (FGF), and insulin-like growth factor-1 (IGF-1) have been shown to play critical roles in wound healing and epithelial renewal [37].

EGF and PDGF are known to stimulate the proliferation and migration of basal epithelial cells, while FGF and IGF-1 support cellular differentiation and enhance structural repair. TGF-β, although more pleiotropic, contributes to epithelial homeostasis by regulating extracellular matrix remodeling and by modulating the local inflammatory response. Together, these factors act in synergy to promote the regeneration of a functional epithelium [38]. One of the earliest observable effects of PRP in DED patients is the improvement in epithelial integrity. Clinically, this is often reflected by reductions in punctate epithelial erosions and improved fluorescein staining patterns. These outcomes are supported by histological and confocal observations showing enhanced epithelial stratification and more regular cell architecture following treatment. Additionally, epithelial cell junctions appear to be reinforced, as suggested by increased expression of tight junction proteins such as ZO-1 and occludin in experimental models [39,40].

PRP may also contribute to a reduction in epithelial apoptosis. This effect is likely linked to the presence of survival-promoting cytokines and anti-apoptotic growth factors within the platelet secretome. In environments of hyperosmolar or oxidative stress—conditions typical of chronic DED—these molecules help protect epithelial cells from damage and preserve the regenerative potential of the ocular surface [41]. A secondary benefit of improved epithelial structure is the stabilization of the tear film. The ocular surface epithelium, particularly the microvilli of the apical cells, provides the anchoring substrate for mucins, which are essential for tear film spreading and retention. By restoring epithelial morphology and promoting mucin gene expression (such as MUC1 and MUC16), platelet-based therapies may help re-establish the glycocalyx, thereby improving tear film quality and reducing evaporation [42,43].

Although the direct stimulation of goblet cell proliferation by PRP remains unclear, the reduction in local inflammation may indirectly support goblet cell function and survival. This is especially relevant in cases of severe or chronic inflammation, where goblet cell loss is a key driver of tear film instability and ocular surface discomfort [44]. Furthermore, in specific DED subtypes—such as those following refractive or cataract surgery, or those with neurotrophic components—PRP appears to influence corneal nerve regeneration. Sub-basal nerve plexus damage is common in these conditions and is associated with reduced reflex tearing, impaired epithelial feedback, and slower wound healing. Platelet-derived factors, including nerve growth factor (NGF) and brain-derived neurotrophic factor (BDNF), may help promote axonal regeneration and improve sensory function. Clinically, this may translate into improved tear production, enhanced corneal sensitivity, and better surface hydration [45,46].

Overall, platelet-based therapies exert their beneficial effects on the ocular surface through multiple converging pathways (Table 2): stimulating epithelial proliferation, reinforcing cell junctions, protecting against apoptosis, supporting neurotrophic repair, and promoting a more stable tear film. This multifactorial approach offers a biologically plausible mechanism for their observed efficacy in managing moderate-to-severe cases of dry eye disease, particularly when conventional therapies prove insufficient.

### 3.3. Clinical Applications of Platelet-Derived Therapies in Dry Eye Disease

This analysis delineates and distinguishes the primary platelet-derived formulations utilized in dry eye disease (DED) prior to examining clinical outcomes. Platelet-rich plasma (PRP) is characterized as an autologous plasma fraction with a platelet concentration exceeding that of baseline whole blood, potentially containing varying quantities of leukocytes based on the preparation process (leukocyte-poor versus leukocyte-rich PRP). Plasma rich in growth factors (PRGF) is characterized as a standardized, leukocyte-depleted plasma fraction acquired through single centrifugation and controlled activation, yielding a consistent quantity of platelet-derived growth factors with minimal pro-inflammatory cytokines. Platelet lysate is described as the supernatant acquired following many freeze–thaw cycles or chemical activation of platelet concentrates, resulting in full platelet degranulation and the release of intracellular growth factors, with minimal remaining cells. Autologous serum tears (ASTs) are defined as the cell-free serum fraction derived from clotted peripheral blood, containing epitheliotropic substances such as epidermal growth factor (EGF), vitamin A, and fibronectin, devoid of concentrated platelets. Consequently, these formulations have not been regarded as interchangeable, and their unique composition, leukocyte content, and growth factor profiles have been delineated in the comprehensive comparison in Table 3.

In recent years, the use of platelet-derived therapies has expanded considerably in the clinical management of moderate-to-severe DED, particularly in patients who fail to respond to conventional treatments. These offer a therapeutic approach that integrates anti-inflammatory, regenerative, and lubricating properties into a single intervention [47]. Clinically, patients treated with PRP-based eye drops have shown consistent improvement in both objective and subjective markers of disease severity. These include increased tear film break-up time, reduced corneal fluorescein staining, and improved Schirmer test scores. Symptomatically, reductions in dryness, burning, photophobia, and foreign body sensation are commonly reported within the first weeks of treatment [48]. Improvements tend to be more pronounced in patients with severe forms of the disease, including those with Sjögren’s syndrome, neurotrophic keratopathy, or post-surgical dry eye [49].

Multiple studies using in vivo confocal microscopy have documented improvements in cell morphology and a reduction in inflammatory cell density following treatment with PRP [50]. These findings are often accompanied by decreased ocular surface inflammation, enhanced epithelial healing, and increased goblet cell preservation. While the precise mechanisms underlying these outcomes are complex, they are likely due to the synergistic action of growth factors, anti-apoptotic peptides, and matrix remodeling enzymes naturally present in platelet-rich formulations [51]. Among available products, PRGF—obtained through selective activation and leukocyte depletion—has shown particularly favorable tolerability, with reduced risk of pro-inflammatory cytokine release [52]. In clinical settings, PRGF has demonstrated comparable efficacy to PRP in terms of epithelial regeneration and symptom relief, with some reports noting faster resolution of epithelial defects and improved corneal nerve density [53]. Its use has gained traction particularly in patients with chronic inflammation or ocular surface damage unresponsive to lubricants, corticosteroids, or cyclosporine [54].

Autologous serum tears also remain a widely used option and share many of the same regenerative properties as PRP, including the presence of EGF, vitamin A, fibronectin, and other trophic factors. However, serum formulations generally lack the higher concentrations of platelet-derived molecules present in PRP and may be less effective in stimulating neuroepithelial repair [55]. The frequency and duration of treatment protocols vary across institutions. Most clinical applications involve the use of undiluted or mildly diluted PRP (20–100%) administered four to six times daily over several weeks [34]. In some centers, repeated cycles of PRP application are used for long-term maintenance, especially in patients with autoimmune or neurotrophic etiologies [16,17]. The stability and sterility of these preparations require specialized compounding and cold-chain storage, which can limit accessibility in some healthcare settings.

Adverse effects are rare but may include transient discomfort upon instillation or hypersensitivity reactions in isolated cases. No major safety concerns have been reported in recent clinical series, and long-term tolerance appears favorable, particularly given the autologous nature of the treatment [33,56,57]. Despite growing clinical adoption, challenges remain. There is currently no universal standard for PRP preparation, leading to considerable heterogeneity in concentration, leukocyte content, activation methods, and dosing regimens. This variability complicates the interpretation of clinical outcomes and hinders the development of evidence-based guidelines. Comparative trials directly evaluating PRP against AST, PRGF, and other biologics are needed to increase standardization and usage of these products.

Moreover, recent research indicates that therapy with PRP or PRGF eye drops has yielded significant clinical enhancements, including an average increase in tear break-up time (TBUT) by 3–5 s, a reduction in OSDI scores by roughly 25–45%, and a mean improvement of 2–4 mm in the Schirmer test. Indeed, most of the current evidence originates from prospective cohort studies and non-randomized clinical trials, exhibiting a middling level of methodological quality. A limited number of randomized controlled studies—specifically those conducted by Jongkhajornpong [33], Sachan [34], and Mederle [47]—have substantiated these advantageous effects; nonetheless, bigger multicenter trials are necessary to reinforce the evidence base.

Table 3 summarizes comparative characteristics of the biologic formulations used in dry eye disease (DED).

In the examined literature, adverse events linked to platelet-based eye drops have been infrequent and typically moderate. Of the sixty-one studies analyzed, transitory ocular or local reactions were the predominant category of reported occurrences. These comprised transient burning, stinging, foreign-body sensation, or conjunctival hyperemia, noted in around 5–10% of treated eyes, and generally resolved spontaneously within minutes to hours. No instances of infection, corneal degradation, or vision-threatening irritation were recorded.

Treatment discontinuations have been rare, occurring in less than 5% of patients overall, primarily due to subjective discomfort or the danger of bottle contamination rather than pharmacologic intolerance. No systemic adverse reactions, including allergic responses, fever, or systemic inflammatory events, have been associated with topical PRP, PRGF, or autologous serum therapy.

Upon reporting, investigators categorized these reactions as mild and non-serious based on conventional criteria, with causality assessed as possible or probable in the majority of instances. Cumulative evidence suggests that platelet-derived eye drops are generally well tolerated and safe for both short- and medium-term application; nevertheless, the lack of extensive multicenter trials restricts accurate assessment of infrequent or long-term adverse effects. Subsequent research should include consistent definitions of local and systemic responses, standardized severity grading, and rigorous documentation of discontinuation rates to enable dependable safety comparisons among biologic formulations.

### 3.4. Historical Background and Clinical Evidence

The modern application of platelet-derived preparations in ocular surface disease is moreover supported by the research of Alio and colleagues, who proposed the use of autologous PRP as a treatment modality in ophthalmology. Their preliminary research indicated that PRP eye drops facilitated re-epithelialization and stromal remodeling in patients with quiescent or non-healing corneal ulcers, signifying an advancement in regenerative ophthalmic therapy [58]. Subsequent research by the same group expanded these findings to the management of symptomatic dry eye disease and ocular surface syndrome post-laser-assisted in situ keratomileusis (LASIK), offering preliminary evidence that platelet concentrates may restore epithelial integrity, alleviate ocular discomfort, and enhance tear film stability [59,60].

In addition to their direct clinical benefits, these findings elucidated the biological basis for PRP by highlighting its substantial concentration of growth factors and cytokines that regulate inflammation resolution and tissue regeneration. The research also prompted the creation of standardized formulations, including PRGF and platelet lysate, which now form the basis for the majority of contemporary clinical regimens. Ten years later, Alio et al. released an extensive study encapsulating the knowledge on “Eye Platelet-Rich Plasma” (E-PRP), clarifying its mechanisms in epithelial healing, neuron regeneration, and anti-inflammatory modulation [61]. Their contribution serves as a crucial reference for comprehending the translation of platelet-based biologics into ophthalmic wound-healing therapy.

Consequently, by demonstrating the safety and regeneration potential of PRP, they established the crucial clinical proof-of-concept that has since been enhanced through controlled trials, optimization of platelet activation techniques, and comparison with autologous serum or PRGF formulations. The integration of these foundational studies into the existing framework highlights that the advancement of platelet-based therapy for dry eye disease is a product of a continuous research trajectory, evolving from original empirical applications to contemporary physiologically informed precision methodologies.

### 3.5. Translational Perspectives and Future Directions

The therapeutic potential of platelet-derived products in dry eye disease demonstrates the intersection of immunomodulation and tissue regeneration. Nevertheless, despite encouraging results, numerous deficiencies remain. The biological variability of platelet preparations and the absence of mechanistic studies hinder complete translational integration. Subsequent research should concentrate on platelet-derived extracellular vesicles (PEVs), which serve as vehicles for RNA, lipids, and proteins that can influence ocular surface immunology and epithelial regeneration. Recent research by Burnouf et al. (2025) has shown that these vesicles may constitute stable and bioactive elements of platelet-derived formulations [26].

Moreover, recognizing biomarkers that forecast responses to PRP or PRGF—such as TGF-β1, NGF, or MMP-9 modulation—may inform personalized therapy approaches. A multidisciplinary strategy that combines molecular profiling, imaging biomarkers, and clinical objectives is essential for advancing platelet-based regenerative ophthalmology from empirical practices to standardized, evidence-based applications.

The translational advancement of platelet-derived therapeutics for dry eye disease necessitates coordinated efforts in standardization, validation, and clinical harmonization. The primary objective is to provide standardized and GMP-compliant preparation techniques for platelet-rich plasma (PRP), plasma rich in growth factors (PRGF), platelet lysate, and autologous serum tears. This standardization is crucial for reducing inter-study variability and enabling repeatable characterization of cellular and soluble components, such as platelet counts, leukocyte content, activation methods, and storage conditions. A second crucial domain pertains to the identification of predictive biomarkers that may inform tailored treatment regimens. Levels of transforming growth factor-β1 (TGF-β1), nerve growth factor (NGF), and matrix metalloproteinase-9 (MMP-9) in tears or serum have surfaced as potential indicators of the inflammatory, neurotrophic, and remodeling equilibrium of the ocular surface. The prospective validation of these biomarkers may facilitate the categorization of individuals who are most likely to benefit from platelet-derived formulations. Third, sufficiently powered, multicenter randomized controlled studies are crucial to validate efficacy, durability, and safety, as well as to establish standardized outcomes including tear break-up time, corneal staining scores, and improvements in the Ocular Surface Disease Index. The integration of GMP-based manufacturing and quality control frameworks will be essential to guarantee batch consistency, traceability, and regulatory approval for clinical applications. By implementing these integrated measures, the field may evolve from empirical applications to evidence-based, precision-engineered platelet biologics that could transform the treatment paradigm of dry eye disease.

### 3.6. Critical Evaluation of Clinical Evidence and Bias Risk

Upon thorough appraisal, clinical evidence endorsing platelet-derived therapy in dry eye disease (DED) has been constrained by many methodological deficiencies. The majority of existing research has involved very small cohorts, was undertaken at individual tertiary facilities, and employed non-randomized or uncontrolled methodologies. Participant and outcome assessor masking has seldom been employed, and follow-up periods have often been less than 6–12 months. These characteristics have heightened the possibility of selection, performance, and detection biases, hence limiting the capacity to derive definitive conclusions about long-term efficacy and safety.

Moreover, significant heterogeneity has been observed in the preparation protocols for PRP, PRGF, platelet lysate, and autologous serum tears, encompassing variations in centrifugation speeds, platelet enrichment, leukocyte contamination, activation techniques, dilutions, instillation frequency, and storage conditions. Outcome measurements and thresholds for clinical success have varied significantly between research, with uneven reporting of defined endpoints, including the Ocular Surface Disease Index (OSDI), tear break-up time (TBUT), corneal and conjunctival staining, and in vivo confocal microscopy parameters. Collectively, these constraints indicate that the majority of existing data pertains to level III–IV evidence, with a limited number of randomized controlled trials accessible. Subsequent research must involve stringent randomization and blinding, standardized protocols, and predetermined primary outcomes, preferably within multicenter frameworks that ensure sufficient statistical power and external validity.

Recent advancements have further solidified the immunoregenerative framework endorsing the therapeutic application of blood- and platelet-derived preparations in ocular surface diseases. Posarelli et al. have presented a comprehensive overview of ocular-surface regeneration therapies, highlighting autologous serum, platelet derivatives, and growth-factor-based biologics as essential instruments for restoring epithelial integrity and modulating para-inflammatory mechanisms in dry eye disease and associated ocular surface disorders [62]. Expanding upon these concepts, Gabriel, Burnouf and colleagues have recently introduced the comprehensive term “eye drops of human origin” (EDHO), which includes serum-, platelet-lysate-, and cord-blood-derived formulations. They have underscored the pressing necessity for standardized preparation protocols, GMP-compliant manufacturing processes, and quality-control systems to guarantee safety and reproducibility in clinical applications [63]. These modern analyses augment and expand upon the foundational clinical studies by Alió et al., who initially established that autologous platelet-rich plasma (E-PRP) eye drops markedly enhance tear film stability, diminish corneal staining, and expedite epithelial recovery in post-LASIK ocular surface syndrome, thereby providing essential clinical evidence for platelet-derived biologics in ophthalmology [64]. These contributions collectively support the advancement of standardized, biomarker-driven, and regulatory-compliant platelet-derived formulations as a novel treatment approach in dry eye disease.

## 4. Conclusions

Platelet-derived therapies represent a promising regenerative approach for the management of DED, particularly in patients with moderate-to-severe or refractory forms. By combining anti-inflammatory, trophic, and neurotrophic properties, platelet-rich formulations target key mechanisms of disease, including epithelial barrier disruption, tear film instability, and chronic ocular surface inflammation. Clinical studies consistently report improvements in both symptoms and objective markers, with favorable safety profiles. However, heterogeneity in preparation protocols and treatment regimens remains a major limitation, highlighting the need for standardized methodologies and controlled comparative trials. Platelet-derived preparations have emerged as prospective biological treatments for dry eye disease by integrating anti-inflammatory, trophic, and neurotrophic effects. Their complex secretome offers a biologically based method that connects immune control with epithelium regeneration. The advancement of standardized preparation techniques, biomarker-directed patient selection, and further investigation of platelet-derived vesicles may characterize the forthcoming generation of regenerative ophthalmic therapeutics. The analysis of existing evidence for platelet-derived therapeutics in dry eye disease is limited by methodological inconsistencies among studies. Variations in platelet concentration, activation techniques, storage conditions, and injection frequency lead to variable results. The lack of randomized controlled trials and the prevalence of small, single-center research restrict generalizability. Moreover, variability in clinical endpoints—such as discrepancies in TBUT or OSDI scores—and the absence of uniform follow-up periods hinder data comparison. Accessibility and financial constraints may limit the availability of these therapies in standard practice. Mitigating these limitations by standardized methodologies and collaborative research networks is a crucial step toward enhanced clinical translation.

## Figures and Tables

**Table 1:** Immunoregulatory roles of platelets in dry eye disease.

Mechanism	Platelet-Derived Factors	Effect on Ocular Surface/DED	Model/Evidence Level
Cytokine release	IL-1β, IL-6, IL-10, TGF-β	Balance of inflammatory signaling; NF-κB suppression; Treg activation	In vitro, in vivo
Chemokine and lipid mediators	CXCL4, CCL5, PAF	Immune cell recruitment and trafficking	In vitro, limited in vivo
Suppression of inflammation	IL-10, TGF-β	Downregulation of IL-1β, TNF-α, IL-6, MMP-9	In vitro, in vivo
Immune cell modulation	P-selectin, CD40L	Crosstalk with leukocytes; modulation of antigen presentation	In vitro, extrapolated
Tissue regeneration	EGF, PDGF, VEGF	Epithelial proliferation, wound healing, nerve repair	In vivo, clinical
Extracellular vesicles	mRNA, miRNA cargo	Post-transcriptional regulation of inflammation and repair	In vitro, emerging
Clinical effects	PRP eye drops	Improved TBUT, reduced staining, symptom relief	Clinical (RCTs, meta-analyses)

Abbreviations: DED, dry eye disease; PRP, platelet-rich plasma; PRGF, plasma rich in growth factors; PL, platelet lysate; AST, autologous serum tears; IL, interleukin; TNF-α, tumor necrosis factor alpha; TGF-β, transforming growth factor beta; PDGF, platelet-derived growth factor; MMP, matrix metalloproteinase; NF-κB, nuclear factor kappa-light-chain-enhancer of activated B cells; ROS, reactive oxygen species.

**Table 2:** Mechanisms of epithelial repair and tear film stabilization mediated by platelet-derived therapies in DED.

Mechanism/Effect	Key Platelet-Derived Factors	Biological Outcome on Ocular Surface
Epithelial proliferation and migration	EGF, PDGF	Stimulates basal cell division and wound closure
Cell differentiation and structural repair	FGF, IGF-1	Enhances stratification and epithelial maturation
Extracellular matrix remodeling and homeostasis	TGF-β	Regulates matrix turnover, controls fibrosis, modulates inflammation
Tight junction reinforcement	Occludin, ZO-1 (upregulated)	Restores barrier integrity, reduces permeability
Anti-apoptotic protection	Cytokines, survival factors	Limits epithelial apoptosis under oxidative/hyperosmolar stress
Tear film stabilization	Indirect via epithelial repair, mucin upregulation (MUC1, MUC16)	Improves glycocalyx, reduces evaporation, enhances spreading
Goblet cell support	Anti-inflammatory milieu (IL-10, TGF-β)	Preserves mucin-secreting cells, supports tear film stability
Neurotrophic repair	NGF, BDNF	Promotes sub-basal nerve plexus regeneration, restores reflex tearing
Clinical outcomes	PRP/PRGF formulations	Reduced staining, improved TBUT, better hydration and sensitivity

Abbreviations: PRP, platelet-rich plasma; PRGF, plasma rich in growth factors; PL, platelet lysate; AST, autologous serum tears; EGF, epidermal growth factor; PDGF, platelet-derived growth factor; FGF, fibroblast growth factor; TGF-β, transforming growth factor beta; VEGF, vascular endothelial growth factor; IGF-1, insulin-like growth factor 1; NGF, nerve growth factor; BDNF, brain-derived neurotrophic factor; ECM, extracellular matrix; GMP, Good Manufacturing Practice.

**Table 3:** Comparative characteristics of biologic formulations used in dry eye disease (DED).

Formulation	Source/Preparation Method	Leukocyte Content	Key Growth Factors/Components	Clinical Advantages	Main Limitations	Typical Clinical Outcomes (Reported Ranges)
Platelet-Rich Plasma (PRP)	Autologous whole blood; single or double centrifugation; activated with calcium chloride or mechanical agitation	Variable (often present)	PDGF, EGF, VEGF, TGF-β, NGF, FGF, IGF-1, serotonin	High concentration of trophic and neurotrophic factors; promotes epithelial regeneration and nerve repair; anti-inflammatory and lubricating properties	Variable preparation protocols; possible mild irritation due to residual leukocytes; short shelf life	↑ TBUT by 3–5 s; ↓ OSDI by 25–45%; improved epithelial staining and patient comfort
Plasma Rich in Growth Factors (PRGF)	Autologous blood; standardized single centrifugation; leukocyte-depleted and activated under controlled conditions	Minimal to absent	PDGF, EGF, IGF-1, TGF-β, VEGF	High reproducibility and safety; reduced pro-inflammatory cytokines; excellent tolerability; promotes epithelial healing	Requires specific proprietary equipment; slightly lower neurotrophic content than PRP	↑ TBUT by 4–6 s; ↓ OSDI by 30–50%; marked improvement in epithelial integrity
Autologous Serum Tears (AST)	Clotted autologous blood; centrifuged to obtain serum fraction; no platelet concentration	None	EGF, Vitamin A, Fibronectin, TGF-β, Albumin	Easy preparation; cell-free; well tolerated for long-term use	Lacks platelet-specific growth factors; lower regenerative potency; limited efficacy in severe or neurotrophic DED	↑ TBUT by 2–3 s; ↓ OSDI by 15–25%; modest improvement in tear stability and staining

Abbreviations: DED, dry eye disease; PRP, platelet-rich plasma; PRGF, plasma rich in growth factors; PL, platelet lysate; AST, autologous serum tears; TBUT, tear break-up time; OSDI, Ocular Surface Disease Index; RCT, randomized controlled trial; GMP, Good Manufacturing Practice; QoL, quality of life; PEVs, platelet-derived extracellular vesicles; ↑ increased; ↓ decreased.

## Data Availability

No new data were created or analyzed in this study.
